# In vivo toxicity evaluation of tumor targeted glycol chitosan nanoparticles in healthy mice: repeated high-dose of glycol chitosan nanoparticles potentially induce cardiotoxicity

**DOI:** 10.1186/s12951-023-01824-3

**Published:** 2023-03-09

**Authors:** Hyeyoun Chang, Ji Young Yhee, Sangmin Jeon, Man Kyu Shim, Hong Yeol Yoon, Sangmin Lee, Kwangmeyung Kim

**Affiliations:** 1 Medicinal Materials Research Center, Biomedical Research Division, Korea Institute of Science and Technology, Seoul, 02792 Republic of Korea; 2 Department of Pharmacy, College of Pharmacy, Kyung Hee University, Seoul, 02447 Republic of Korea; 3 College of Pharmacy, Graduate School of Pharmaceutical Sciences, Ewha Womans University, Seoul, 03760 Republic of Korea

**Keywords:** Glycol chitosan nanoparticles, Nanotoxicology, Toxicity evaluation, Cardiotoxicity

## Abstract

**Background:**

Glycol chitosan nanoparticles (CNPs) have emerged as an effective drug delivery system for cancer diagnosis and treatment. Although they have great biocompatibility owing to biodegradable chemical structure and low immunogenicity, sufficient information on in vivo toxicity to understand the potential risks depending on the repeated high-dose have not been adequately studied. Herein, we report the results of in vivo toxicity evaluation for CNPs focused on the number and dose of administration in healthy mice to provide a toxicological guideline for a better clinical application of CNPs.

**Results:**

The CNPs were prepared by conjugating hydrophilic glycol chitosan with hydrophobic 5β-cholanic acid and the amphiphilic glycol chitosan-5β-cholanic acid formed self-assembled nanoparticles with its concentration-dependent homogeneous size distributions (265.36–288.3 nm) in aqueous condition. In cell cultured system, they showed significantly high cellular uptake in breast cancer cells (4T1) and cardiomyocytes (H9C2) than in fibroblasts (L929) and macrophages (Raw264.7) in a dose- and time-dependent manners, resulting in severe necrotic cell death in H9C2 at a clinically relevant highly concentrated condition. In particular, when the high-dose (90 mg/kg) of CNPs were intravenously injected into the healthy mice, considerable amount was non-specifically accumulated in major organs (liver, lung, spleen, kidney and heart) after 6 h of injection and sustainably retained for 72 h. Finally, repeated high-dose of CNPs (90 mg/kg, three times) induced severe cardiotoxicity accompanying inflammatory responses, tissue damages, fibrotic changes and organ dysfunction.

**Conclusions:**

This study demonstrates that repeated high-dose CNPs induce severe cardiotoxicity in vivo. Through the series of toxicological assessments in the healthy mice, this study provides a toxicological guideline that may expedite the application of CNPs in the clinical settings.

**Supplementary Information:**

The online version contains supplementary material available at 10.1186/s12951-023-01824-3.

## Introduction

In the field of medicine, nanotechnology has shown great potential as a means for targeted drug delivery in cancer treatment [1–3]. A variety of nanoparticles, such as polymeric nanoparticles, liposomes, dendrimers, micelles and inorganic nanoparticles, have been developed as an innovative delivery systems of contrast agents and chemical drugs for cancer diagnosis and treatment [4–6]. The leaky structure of angiogenic blood vessels and dysfunctional lymphatic systems in tumors provide a favorable environment for nanoparticles to accumulate within the tumor tissues and become retained at the site [7–10]. Based on this enhanced permeability and retention (EPR) effect, nanoparticles demonstrate considerable tumor accumulation, which makes a suitable candidate for tumor-targeting strategy [11–15]. However, current negative consequence for clinical translation of nanoparticles is unexpectedly low tumor-targeting efficiency, with only about 1% of the administered nanoparticles being reached to tumor tissues [16]. In addition, tumors in patients represent highly heterogeneous and complex features than the experimental tumors in animal models; thus even nanoparticles showing excellent delivery efficiency in animal models eventually failed in targeting the tumor tissues of patients [17].

Such negative consequence of low tumor-targeting efficiency of nanoparticles is directly related to manufacturing, drug price, therapeutic efficacy and especially toxicity [18]. Although many studies on biodegradable organic polymer-based nanoparticles have showed relatively low cytotoxicity in vitro, sufficient information on in vivo toxicity to understand the potential risks of nanoparticles have not been adequately reported [19]. The reason why these studies are important is because drug loading capacity of nanoparticles are less than 10wt%; therefore, 10 times more amount of carrier materials than anticancer drugs is simultaneously administered in the body [20–23]. For example, FDA-approved liposomal doxorubicin, DOXIL vial contains 50 mg of doxorubicin, which is treated at a recommended dose of 50 mg/m^2^ based on doxorubicin contents without any mention of the equivalent dose of materials for liposome formulation [24]. However, there are the risk of potential toxicity because approximately 500 mg of carrier materials inevitably administered in vivo. Hence, biocompatibility and in vivo toxicity by excess amount and repeated dose of nanoparticles should be studied to provide toxicological guideline for clinical use of nanoparticles.

**Scheme 1.**
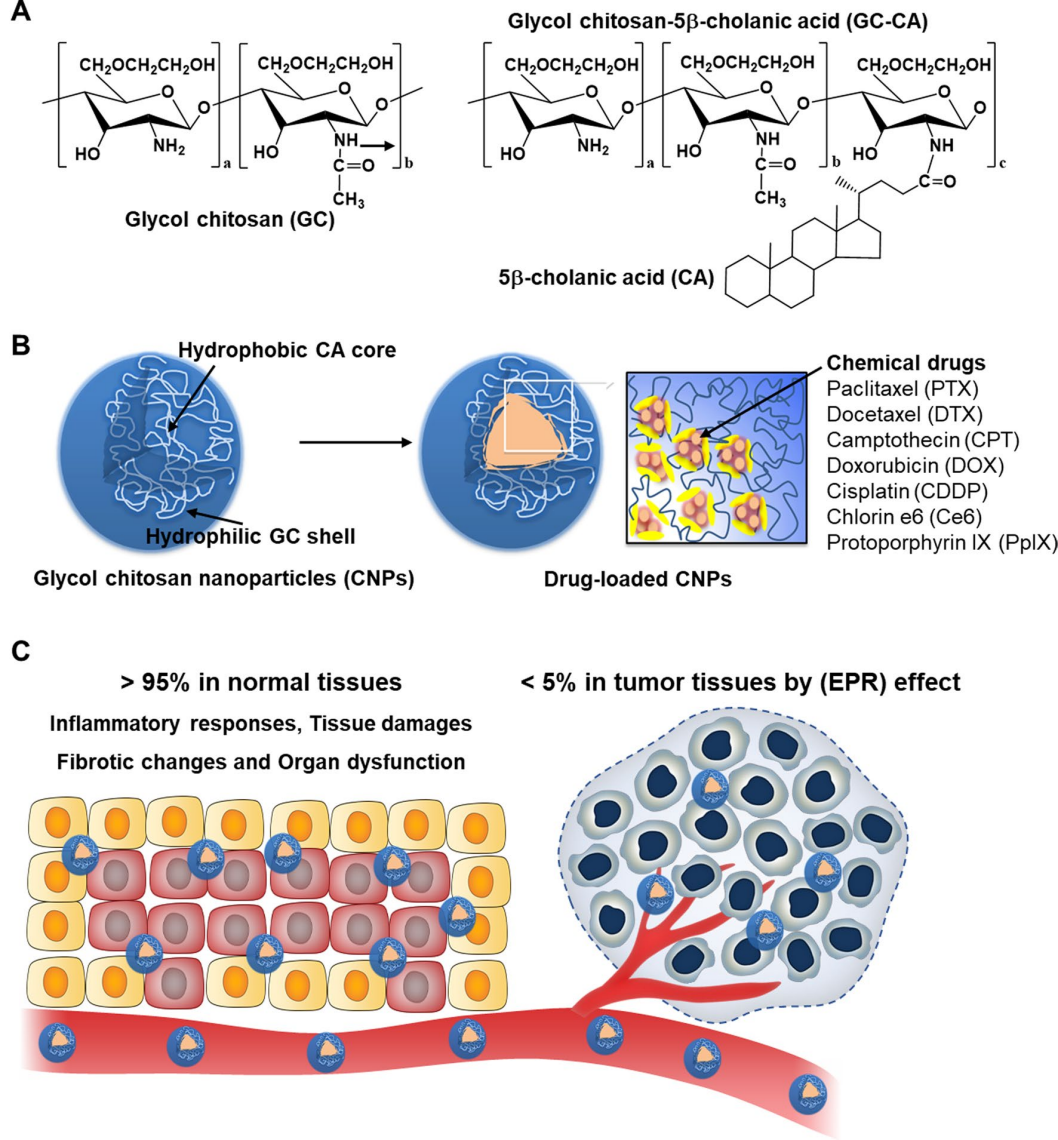
In vivo toxicity evaluation of glycol chitosan nanoparticles (CNPs).** A** The CNPs were prepared by conjugating hydrophilic glycol chitosan with hydrophobic 5β-cholanic acid.** B** These CNPs have been widely studied for tumor-targeted delivery of various chemical drugs, such as paclitaxel (PTX), docetaxel (DTX), camptothecin (CPT), doxorubicin (DOX), cisplatin (CDDP), chlorin e6 (Ce6) and protoporphyrin IX (PpIX). **C** When the CNPs were intravenously injected into tumor models, although they can efficiently accumulate within the tumor tissues by EPR effect, tumor-targeting efficiency is less than 5% and considerable amount (> 95%) is non-specifically accumulated in the normal organs. The precise toxicological risks caused by these in vivo behaviors of CNPs have not been fully investigated at repeated high-dose condition; thus, we performed the in vivo toxicity evaluation for CNPs focused on the number and dose of administration to provide a toxicological guideline for a better clinical application of CNPs

Recently, chitosan polymers or its derivatives have been widely utilized in formulating nanoparticles over the last few decades owing to its high biodegradability, biocompatibility and low immunogenicity [25]. In particular, glycol chitosan nanoparticles (CNPs), which are prepared by conjugating hydrophobic 5β -cholanic acid to hydrophilic glycol chitosan, have been extensively used in targeted drug delivery for cancer diagnosis and treatment (Scheme 1A) [26]. The CNPs have been studied for tumor-targeted delivery of various chemical drugs, such as paclitaxel (PTX), docetaxel (DTX), camptothecin (CPT), doxorubicin (DOX), cisplatin (CDDP), chlorin e6 (Ce6) and protoporphyrin IX (PpIX); because these are efficiently loaded into the inner hydrophobic 5β -cholinic acid cores with about 10% of uniform drug loading capacity via simple dialysis methods (Scheme 1b) [27–33]. Importantly, although CNPs can efficiently accumulate within the tumor tissues by EPR effect, tumor-targeting efficiency is less than 5% and considerable amount (> 95%) is non-specifically accumulated in the normal organs (Scheme 1c) [34]. The safety of CNPs have been demonstrated in vitro and in vivo, but their precise toxicological risks have not been fully investigated at repeated high-dose condition. Herein, we report the result of in vivo toxicity evaluation for CNPs focused on the number and dose of administration. First, cellular uptake and cytotoxicity of CNPs is evaluated in breast cancer cell, and three types of normal cells including cardiomyocytes, fibroblasts and macrophages. Furthermore, biodistribution of CNPs at the low- and high-dose is analyzed in the healthy mice. Finally, in vivo toxicity of CNPs along to the repeated high-dose is carefully studied via hematological and histological analyses. This study aims to understand the extensive toxicological features of CNPs and will contribute to a better clinical application of CNPs by providing a toxicological guideline.

## Results and discussion

### Preparation and characterization of glycol chitosan nanoparticles (CNPs)

**Fig. 1:**
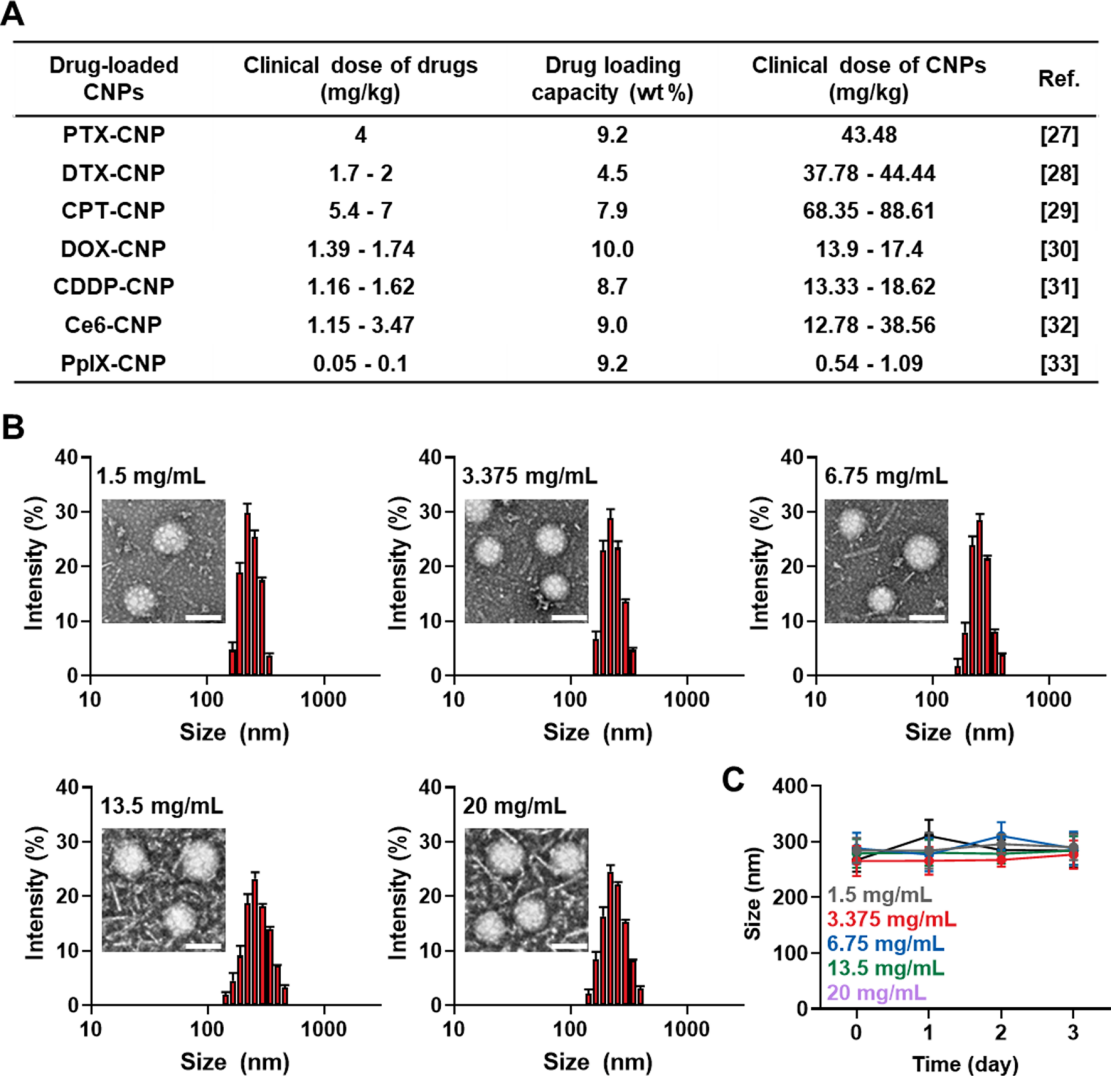
Preparation and characterization of CNPs. **A** The each drug, loadng capacity, injection dose, of the drug-loaded CNPs in previous studies. PTX; paclitaxel, DTX; docetaxel, CPT; camptothecin, DOX; doxorubicin, CDDP; cisplatin, Ce6; chlorin e6 and PpIX; Protophorphyrin IX. **B** Size distribution and morphology of CNPs in the clinically relevant highly concentrated aqueous condition (1.5, 3.375, 6.75, 13.5 or 20 mg/1 ml). The scale bar in TEM images indicates 200 nm (n=5).** C** Size stability of different concentrations of CNPs in the mouse serum (n=5)

Glycol chitosan nanoparticles (CNPs) were successfully prepared by conjugating hydrophobic 5β-cholanic acid (CA) to hydrophilic glycol chitosan polymer (GC) in the in the presence of chemical catalysts, wherein 150 ± 4.5 molecules of CA are conjugated into each GC polymer (Additional file 1: Fig. S1) [35]. The synthesized amphiphilic CA-GC conjugates self-assemble into spherical nanoparticles, which consisted of hydrophilic GC shell and hydrophobic CA core, with a diameter of approximately 260 nm in aqueous condition [36]. It has been also reported that hydrophobic chemical drugs, such as PTX [27], DTX [28], CPT [29], DOX [30], CDDP [31], Ce6 [32] and PpIX [33], could be efficiently loaded into hydrophobic cores in CNPs via a simple dialysis method, showing almost 5–10 wt% drug loading capacity (Fig. 1A). Notably, anticancer drug-loaded CNPs exhibited efficient passive tumor accumulation by in vivo long circulation and could penetrate deep inside the tumor tissues owing to their great stability and deformability in tumor-bearing mice [37]. According to the therapeutic dose of the loaded drugs in CNPs, the expected administration dose of CNPs for preclinical and clinical studies can be estimated; for example, PTX-loaded glycol chitosan should be administered at a dose of 43.48 mg/kg for expecting enough therapeutic efficacy. Accordingly, administration dose of drug-loaded CNPs can be varied along to each anticancer drug (DTX, 44.44 mg/kg; CPT, 88.61 mg/kg; DOX, 17.4 mg/kg; CDDP, 18.62 mg/kg; Ce6, 38.56 mg/kg; and PpIX, 1.09 mg/kg). In this context, we can estimate the in vivo toxicity of repeated high-dose of CNPs up to 90 mg/kg of CNPs that is higher than the clinical dose of CNPs actually used in clinical test. Therefore, we first investigated the size distribution of CNPs in the clinically relevant highly concentrated aqueous condition. Importantly, CNPs showed spherical morphology and homogenous size distribution with average size of 265.36–288.3 nm when 1.5, 3.375, 6.75, 13.5 or 20 mg were dispersed in 1 mL saline, showing uniform particle size even at the extremely high concentration (Fig. 1B). There was no significant difference in zeta potential (mV) values of CNPs according to the concentration (Additional file 1: Fig. S2). These results verify that CNPs can maintain their particle structure in the clinically relevant highly concentrated aqueous condition for preclinical and clinical study. In addition, CNPs in different concentration (1.5, 3.375, 6.75, 13.5 or 20 mg/mL) showed excellent stability in mouse serum without significant changes in particle sizes for 3 days (Fig. 1C and Additional file 1: Fig. S3). This is because the CNPs have great stability against serum nucleases [35]. From these findings, it is expected that CNPs can be used at high concentration without changes of their intrinsic nanoparticle structure.

### Cellular uptake and cytotoxicity of CNPs in cancer and normal cells

**Fig. 2:**
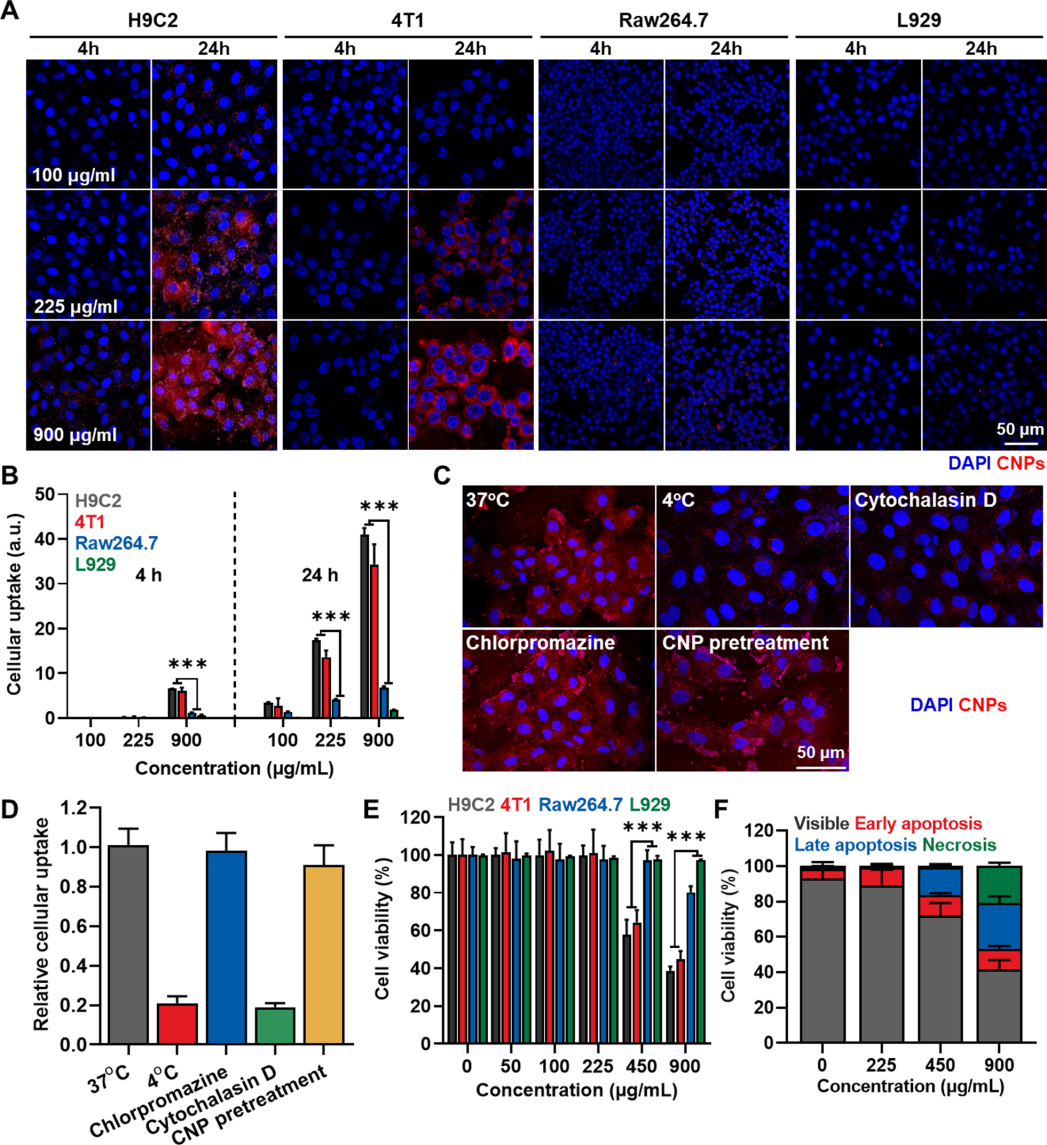
Cellular uptake and cytotoxicity of CNPs. **A** Cellular uptake of CNPs in 4T1 breast cancer cells, H9C2 cardiomyocytes, L929 fibroblast and Raw264.7 macrophages in the condition of different dose and incubation time. **B** Quantitative analysis for the amount of cellular uptake of CNPs in 4T1, H9C2, L929 and Raw264.7 cells in the condition of different dose and incubation time (n = 5). **C** Cellular uptake of CNPs in the H9C2 cells at different temperature and after pretreatment of chlorpromazine or cytochalasin D. **D** Quantitative analysis for cellular uptake of CNPs in the H9C2 cells at different temperature and after pretreatment of chlorpromazine or cytochalasin D (n = 5). **E** The viability of 4T1, H9C2, L929 and Raw264.7 cells after treatment with CNPs for 24 h (n = 5). **F** Annexin V/PI staining of H9C2 cells after treatment with CNPs for 24 h (n = 5)

The cellular uptake and cytotoxicity of CNPs were assessed in breast cancer cell (4T1), and three types of normal cells, including rat cardiomyocytes (H9C2), mouse fibroblasts (L929) and macrophages (Raw264.7). For subcellular tracking, the CNPs were further modified with near-infrared fluorescent dye, Cy5.5, with an average of 4.8 ± 0.7 molecules per CA-GC conjugates. In previous study, it was confirmed that modification with Cy5.5 is not influence on physicochemical and biocompatible characteristics of CNPs [38, 39]. When the Cy5.5-labeled CNPs (Cy5.5-CNPs) were incubated with 4T1 cells and H9C2 cells, only weak fluorescence signals were observed intracellularly at 4 h post-incubation at the concentration from 100 to 900 μg/mL, whereas those of Cy5.5-CNPs were greatly increased after 24 h of incubation at the high concentration of 225 and 900 μg/mL (Fig. 2A). In addition, cellular uptake of Cy5.5-CNPs were gradually increased in a dose-dependent manner, wherein fluorescence signals of Cy5.5-CNPs in 4T1 cells and H9C2 cells got stronger along to the increase of treatment concentration. In contrast, only a little cellular uptake of Cy5.5-CNPs was observed in Raw264.7 and L929 cells even the treatment with high concentrations (900 μg/mL) for 24 h. Quantitatively, cellular uptake of Cy5.5-CNPs after 24 h incubation with 900 μg/mL dose was approximately 25.11–26.3-fold and 6.22–7.12-fold higher in H9C2 and 4T1 cells than in L929 and Raw264.7 cells, respectively (Fig. 2B). The endocytic pathway of CNPs was further assessed to investigate the mechanism for their high cellular uptake to the cardiomyocytes than other normal cells (Fig. 2C). First, the cellular uptake of Cy5.5-CNPs in H9C2 cells was significantly decreased at 4 °C compared to 37 °C, indicating that CNPs primarily enter through an energy-dependent process. In addition, we further assessed the endocytosis pathway of CNPs by measuring the effect of the endocytosis inhibitors on the cellular uptake of nanoparticles. The pretreatment of chlorpromazine, an inhibitor of clathrin-mediated endocytosis, did not prevent the cellular uptake of Cy5.5-CNPs into H9C2 cells compared to naive cells. In contrast, the cellular uptake of Cy5.5-CNPs in the H9C2 cells was greatly inhibited after pretreatment with cytochalasin D that is inhibitor of macropinocytosis. In addition, the pretreatment of CNPs (not labelled with Cy5.5) did not decrease the cellular uptake of Cy5.5-CNPs that was subsequently treated; these results demonstrate that there are no specific receptors related to the cellular uptake of CNPs in the cardiomyocytes. Taken together, these in vitro results clearly demonstrate that CNPs are mainly taken up in cardiomyocytes via micropinocytosis that is well known to be a main cellular uptake mechanism of nanoparticles in the cardiac cells [40]. In agreement with the cellular uptake results, severe cytotoxicity of Cy5.5-CNPs was observed in H9C2 and 4T1 cells compared to Raw264.7 and L929 cells, wherein the cell viability of H9C2 and 4T1 cells was decreased to the levels of about 40% and 60% after 24 h of treatment with 450 and 900 μg/mL of Cy5.5-CNPs, respectively (Fig. 2C). To evaluate the potential toxicity of CNPs in the normal cells that show high cellular uptake, we additionally performed Annexin V/PI staining of H9C2 cells after 24 h of treatment with 900 μg/mL of Cy5.5-CNPs to more clearly assess the cell death patterns after treatment with high concentration of CNPs. Interestingly, only a small quantity of necrosis (0.5 ± 0.09%) and high early (11.5 ± 1.3%) and rate (16 ± 1.7%) apoptosis were observed in H9C2 cells after 24 h of treatment with 450 μg/mL of Cy5.5-CNPs (Fig. 2D and Additional file 1: Fig. S4). Notably, necrotic cell death (20.7 ± 1.98%) of H9C2 cells was greatly increased after treatment of 900 μg/mL Cy5.5-CNPs than 450 μg/mL. The cells undergoing necrosis promote various inflammatory cytokines, such as TNF-α, IL1b and IL6; thus, these results indicate that long-term exposure with high concentration of CNPs can potentially induce the cardiotoxicity in vivo by severe inflammatory responses [41–43].

### In vivo biodistribution of intravenously injected CNPs in healthy mice

**Fig. 3:**
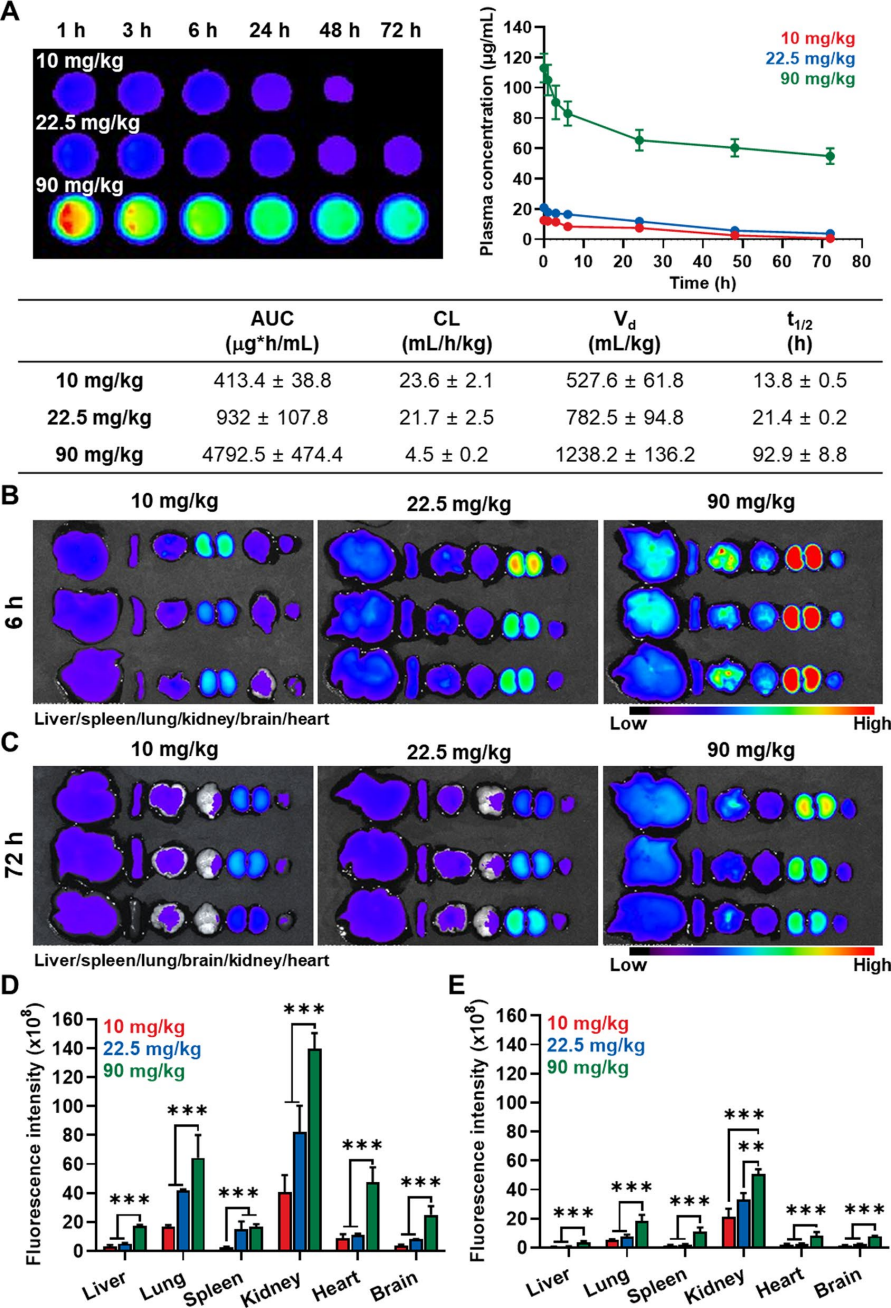
Biodistribution of CNPs in healthy mice. **A** Pharmacokinetic (PK) profiles of CNPs in healthy mice after 10, 22.5 or 90 mg/kg injection (n = 3). **B**, **C** Pharmacodynamic (PD) profiles of CNPs in healthy mice after 10, 22.5 or 90 mg/kg injection. The fluorescence imaging of major organs was performed after **B** 6 h and **C** 72 h of injection. **D**, **E** Quantitative analysis for the amount of CNPs in the major organs after **B** 6 h and **C** 72 h of injection

Next, the pharmacokinetic/pharmacodynamic (PK/PD) profiles of CNPs in the low- and high-dose were assessed in healthy BALB/c mice. To evaluate the PK profiles, three different doses of Cy5.5-CNPs (10, 22.5 and 90 mg/kg) were intravenously injected into the mice, and blood samples were collected at pre-determined time points. Interestingly, Cy5.5-CNPs in bloods were detected up to 48 h of injection with a half-life (*t*_1/2_) of 13.8 ± 0.5 h in mice treated with 10 mg/kg of Cy5.5-CNPs, whereas a detectable amount of Cy5.5-CNPs remained for 72 h in the body with *t*_1/2_ of 21.4 ± 0.2 h when the administered dose was increased to 22.5 mg/kg (Fig. 3A). Notably, 90 mg/kg of Cy5.5-CNPs showed greatly prolonged *t*_1/2_ of 92.9 h, wherein the area under the curves (AUC) was also increased 12.4–12.64-fold and 5.2–5.27-fold compared to 10 or 22.5 mg/kg of Cy5.5-CNPs, respectively. These results indicate that considerable amount of CNPs are remained in the body for a long time along to the dose increase, which can potentially induce in vivo toxicity. Next, PD profiles after treatment with low- or high-dose of Cy5.5-CNP were evaluated by analyzing accumulation in major organs via ex vivo fluorescence imaging. As expected, only a small quantity of Cy5.5-CNPs was observed in the major organs (liver, spleen, lung, kidney, brain and heart) after 6 h of injection with 10 mg/kg, but considerable amount was accumulated in those organs in mice treated with 22.5 or 90 mg/kg Cy5.5-CNPs (Fig. 3B). In addition, while most of Cy5.5-CNPs was removed from the body after 72 h of injection when the dose of 10 or 22.5 mg/kg was administered, those highly accumulated in major organs after 90 mg/kg injection were sustainably retained (Fig. 3C). The Cy5.5-CNPs accumulated in the lung, liver or kidney tissues were significantly higher than those in the heart tissue after 6 h or 72 h of 90 mg/kg injection. Since more than 50% of Cy5.5-CNPs was still remained in the blood after 72 h of 90 mg/kg injection as shown in Fig. 3A, a strong fluorescence of Cy5.5-CNPs was observed in lung, liver or kidney tissues because those organs have many capillaries. The actual amount of Cy5.5-CNPs accumulated in the lung, liver or kidney tissues was further assessed by performing ex vivo imaging of major organs 7 days after most of nanoparticles is excreted from body (Additional file 1: Fig. S5). The result clearly showed that Cy5.5-CNPs in lung, liver or kidney tissues were significantly reduced, whereas similar amount of CNPs were still observed in the heart tissue compared to 72 h after injection. This result indicates that a strong fluorescence of Cy5.5-CNPs in the lung, liver or kidney tissues of ex vivo imaging is what appears from the blood stream of the many capillaries in those organs. As a control, we also found that a significant accumulation of Cy5.5-CNPs in the lymph nodes, bone marrow, thymus and intestinal tract was not observed after 7 days of 90 mg/kg treatment. Quantitative analysis of ex vivo imaging results clearly showed that mice treated with 90 mg/kg Cy5.5-CNPs exhibited significantly higher accumulation in major organs compared to 10 or 22.5 mg/kg Cy5.5-CNPs after 6 h (all the organs: P < 0.001, in comparison to 10 and 22.5 mg/kg Cy5.5-CNPs groups) or 72 h (liver, lung, spleen, heart and brain: P < 0.001, in comparison to 10 and 22.5 mg/kg Cy5.5-CNPs groups; kidney: P < 0.001 and P < 0.05, in comparison to 10 and 22.5 mg/kg Cy5.5-CNPs groups, respectively) of injection (Fig. 3D, E). Taken together, these results demonstrate that non-specific accumulation of CNPs in major organs can be considerably increased and sustainably retained in the body with increasing the administration dose of CNPs.

### Organ toxicity after single-/multi-dose of CNPs in healthy mice

**Fig. 4:**
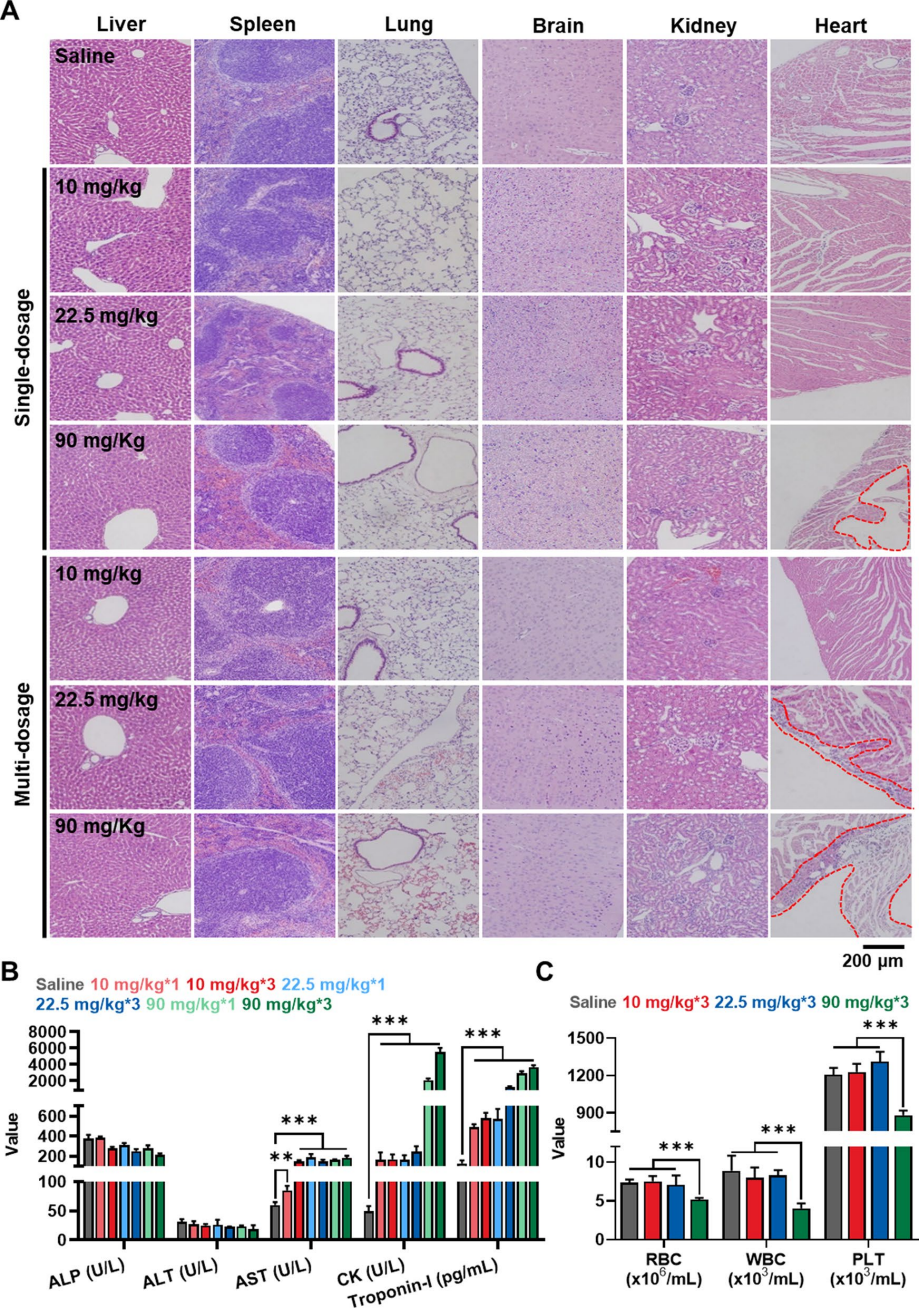
Organ toxicity after single-/multi-dose of CNPs in healthy mice. **A** The major organs stained with H&E after single- or multi-dose of 10, 22.5 or 90 mg/kg CNPs. Histological analyses were performed on day 7 after treatments. **B** Hematological parameters after single- or multi-dose of 10, 22.5 or 90 mg/kg CNPs. Blood samples were collected on day 7 after treatments (n = 5). **C** Complete cell count results after multi-dose of 10, 22.5 or 90 mg/kg CNPs (n=5)

The in vivo toxicity of CNPs was evaluated in the BALB/c mice via histological and hematological analyses after single-/multi-dose. For these analyses, healthy mice were divided into seven groups; (i) saline; (ii) 10 mg/kg single-dose; (iii) 10 mg/kg multi-dose (10 mg/kg*3); (iv) 22.5 mg/kg single-dose; (v) 22.5 mg/kg multi-dose (22.5 mg/kg*3); (vi) 90 mg/kg single-dose; (vii) 90 mg/kg multi-dose (90 mg/kg*3). In the case of the multi-dose groups, mice were treated with Cy5.5-CNPs three times with 2 days-intervals. After 7 days of treatment, the histopathological changes in the organs stained with H&E were observed under microscopy (Fig. 4A). The results exhibited that no significant toxicity-related lesions in the spleen, lung, brain and kidney tissues in all groups. Even though CNPs highly accumulated in the kidney after 6 h of 90 mg/kg treatment as shown in Fig. 3B, a notable toxicity was not observed up to 72 h. In order to explain the higher accumulation of CNPs in kidney, we assessed the excretion profile of Cy5.5-labeled CNPs-treated mice (Additional file 1: Fig. S6). After the CNPs (90 mg/kg) were injected, large amount of CNPs were excreted in the urine from 3 to 9 h, indicating the strong fluorescence signal of Cy5.5-labeled CNPs in kidney at 6 h post-injection is obviously caused by the excretion of enzymatically degraded CNPs through kidney [44]. Then, the fluorescence intensity of Cy-5.5-labeded CNPs in kidney greatly decreased after 72 h post-injection. From the excretion profile of intravenously injected CNPs, the accumulated CNPs in kidney were gradually excreted in the urine and they did not cause notable toxicity in kidney. In contrast, all the groups showed mild hyperplastic changes such as the pale cytoplasm of the hepatocytes in the liver tissues. Most importantly, noticeable tissue damages were not observed in heart tissues from mice treated with 10 or 22.5 mg/kg single-dose, whereas significant structural abnormalities were observed from the heart tissues after 90 mg/kg single-dose. In addition, mice treated with multi-dose of 22.5 or 90 mg/kg showed a degeneration of the heart to a certain degree. In particular, a wide range of pericardial mononuclear cell infiltration was observed in the 90 mg/kg multi-dose group, and calcifications at the pericardium were also observed in the mice. Next, we also performed hematological analyses in the mice treated by same protocol as above (Fig. 4B and Additional file 1: Fig. S7). Firstly, the levels of ALP and ALT were not significantly changed after Cy5.5-CNPs injection in both single- and multi-dose groups compared to saline group. In contrast, the AST levels were in normal ranges after 10 mg/kg single-dose, while those increased significantly in 22.5 and 90 mg/kg single-dose and all the multi-dose groups. These results indicate that repeated high-dose of CNPs treatment result in liver dysfunction. Importantly, the significantly increased levels of hematological parameters related to cardiac function, such as CK and Troponin-I, were clearly observed in 10 and 22.5 mg/kg of single- or multi-dose group; especially, such parameters were more greatly increased in 90 mg/kg of single- and multi-dose groups. In addition, complete blood count results also showed that 90 mg/kg multi-dose of CNPs also resulted in the significant decrease of red blood cells, white blood cells and platelets compared to 10 or 22.5 mg/kg of multi-dose and saline groups (Fig. 4C and Additional file 1: Fig. S8). From these toxicity study, we found that the most obvious side effects that can be induced by repeated high-dose of CNPs treatment are cardiotoxicity, which is accompanied by tissue damages and organ dysfunction.

### Cardiotoxicity evaluation after repeated high-dose of CNPs treatment

**Fig. 5:**
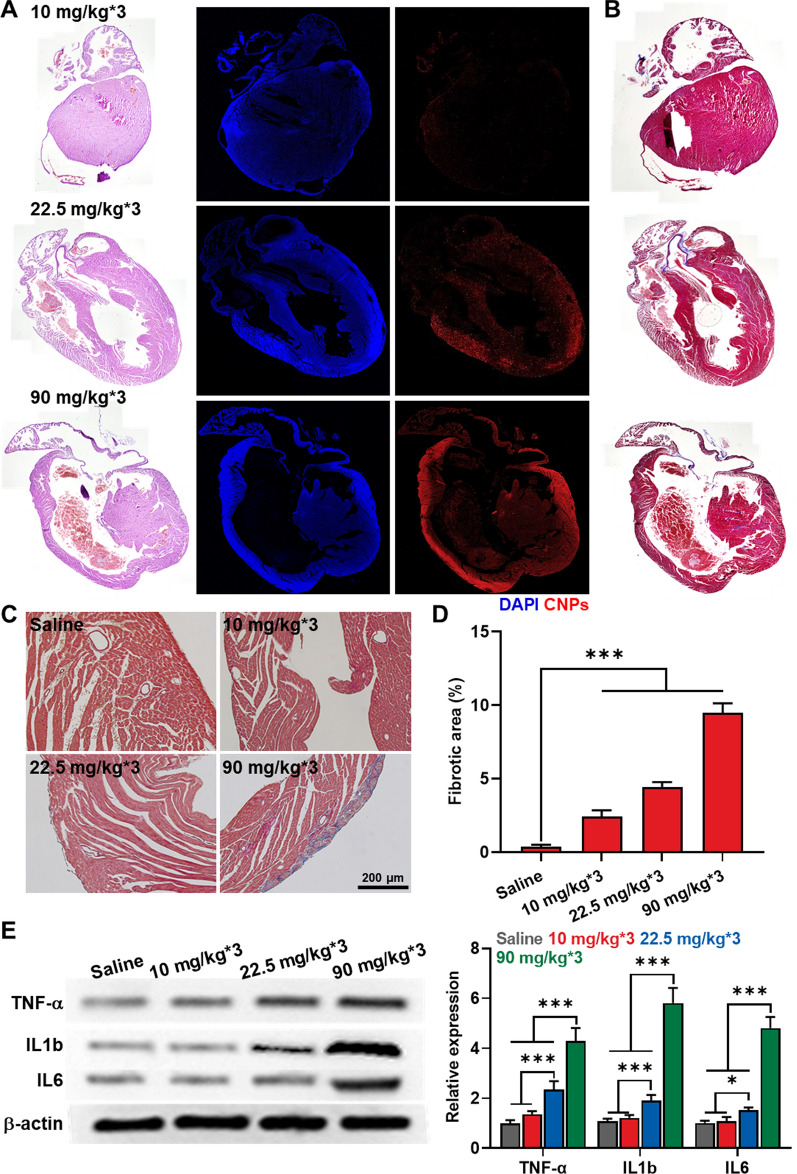
Cardiotoxicity evaluation after repeated high-dose of CNPs treatment. **A** Whole heart tissues stained with H&E after multi-dose of 10, 22.5 or 90 mg/kg CNPs. Right panel indicates the fluorescence imaging of heart tissues after treatments in the same protocol. Histological analyses were performed on day 7 after treatments. **B**, **C** Heart tissues stained with Masson’s trichrome after multi-dose of 10, 22.5 or 90 mg/kg CNPs. **D** Quantative analysis for the fibrotic areas (%) over whole region of heart tissues. Heart tissues were collected from the mice on day 7 after multi-dose of 10, 22.5 or 90 mg/kg CNPs (n = 5). **E** The levles of TNF-α, IL1b and IL-6 in heart tissues on day 7 after multi-dose of 10, 22.5 or 90 mg/kg CNPs

Encouraged by organ toxicity study, we performed additional histology with analysis in molecular levels to assess the cardiotoxicity after repeated high-dose of CNPs treatment at a whole region of heart tissues. Likewise, the mice were treated with 10, 22.5 or 90 mg/kg Cy5.5-CNPs three times with 2 days-intervals, and heart tissues were collected on day 7 after treatment. As expected, detectable fluorescence signals of Cy5.5-CNPs were not observed in whole heart tissues from mice treated with 10 mg/kg multi-dose due to its in vivo clearance, whereas Cy5.5-CNPs were clearly observed after treatment with 22.5 mg/kg multi-dose; notably, those accumulated in whole heart tissues were considerably increased in 90 mg/kg multi-dose group (Fig. 5A). As a result, the mice treated with 22.5 mg/kg or 90 mg/kg multi-dose exhibited mononuclear cell infiltration as well as calcification of the cardiomyocytes in a wide area of whole heart tissues. Although the symptoms of the mice were the same for the 22.5 and 90 mg/kg groups, the degenerations in the heart tissues were severe in the 90 mg/kg group. Next, heart tissues were observed after the Masson’s trichrome staining to evaluate any fibrotic changes (Fig. 5B, C). The multi-dose groups with 22.5 and 90 mg/kg Cy5.5-CNP showed thickened blue-stained pericardia, suggesting chronic pericarditis. In particular, the highest-dose group displayed dense fibrotic tissues stained deep blue around the mononuclear cells, wherein the fibrotic area over whole heart region was significantly increased in the mice treated with 90 mg/kg multi-dose (9.49 ± 0.64%) than 10 (2.42 ± 0.43%) and 22.5 mg/kg (4.44 ± 0.34%) multi-dose (Fig. 5D). Finally, the levels of inflammatory cytokines, including TNF-α, IL1b and IL-6, in heart tissues were measured via western blot analysis (Fig. 5E). This is because high-dose CNPs treatment led to necrotic cell death that promote inflammatory responses in cardiomyocytes as shown in Fig. 2D. The result showed that the levels of inflammatory cytokines in heart tissues from mice treated with 10 mg/kg multi-dose Cy5.5-CNPs were similar with saline group, but repeated high-dose of 22.5 and 90 mg/kg treatments greatly upregulated TNF-α, IL1b and IL-6 in heart tissues. These results indicate that repeated high-dose of CNPs treatment leads to severe inflammatory responses in heart tissues by inducing necrotic cell death. Therefore, in order to apply our CNPs to clinical test, we have to consider the clinical dose of the drug actually used. Based on our study, we can expect that CNPs can be used for drug delivery carrier safely when they are injected with less than 22.5 mg/kg. Considering the maximal drug loading efficiency with 10 wt% of conventional polymeric nanoparticles, CNPs could be used as a reliable nano-sized carrier for anticancer drugs of DOX, CDDP and PpIX that have significant efficacy at clinical doses lower than 2 mg/kg. In addition, the recent great advances in discovering new anticancer drugs based on molecular medicine and gene medicine can greatly expand the opportunity for using the CNPs as a drug delivery carrier in clinic. In this point of view, this study can offer valuable toxicological guidelines for the clinical application of various nano-sized drug carriers.

## Conclusion

In this study, we reported the result of in vivo toxicity evaluation for glycol chitosan nanoparticles (CNPs) focused on the number and dose of administration in healthy mice to provide reliable toxicological data that is crucial for clinical applications. First, we found that CNPs highly accumulated in cardiomyocytes compared to fibroblasts and macrophages, resulting in severe necrotic cell death. This in vitro cytotoxicity study can provide a minimum guideline for safe use of CNPs, but nanoparticles may show unpredictable adverse effects in vivo that include organ toxicity, genotoxicity, and immunotoxicity. Therefore, PK/PD profiles of CNPs in the low- and high-dose were carefully evaluated in healthy mice. The low-dose (10 mg/kg) of CNPs showed relatively fast in vivo clearance within 48 h, whereas high-dose (22.5 and 90 mg/kg) administration resulted in nanoparticle residue for a long-time in body. Hence, mice treated with high-dose of CNPs showed high non-specific accumulation of nanoparticles in major organs, which was sustainably retained with no excretion. As a result, high-dose of CNPs induced severe cardiotoxicity accompanying inflammatory responses, tissue damages, fibrotic changes and organ dysfunction; furthermore, these symptoms were more worsen at the increase of dose number. Through these series of toxicological assessments, this study provides a toxicological guideline that may expedite the application of CNP in the clinical settings.

## Materials and methods

### Reagents

Glycol chitosan (Mw = 250 kDa, 5β-cholanic acid, 1-ethyl-3-(3-dimethylaminopropyl)-carbodiimide hydrochloride (EDC) and N-hydroxysuccinimide (NHS) were purchased from Sigma Aldrich (St. Louis, MO, USA). Flamma 648 NHS ester was purchased from BioActs (Incheon, Republic of Korea). Cell counting kit-8 (CCK-8) was purchased from Vitascientific (Beltsville, MD, USA). Tem grid (Carbon Film 200 Mesh copper) was purchased from Electron Microscopy Sciences (Hatfield, PA, USA). H9C2 (Rat cardiomyocyte), L929 (mouse fibroblast) and Raw264.7 (macrophage) cell lines were purchased from American Type Culture Collection (ATCC; Manassas, VA, USA). Fetal bovine serum (FBS), streptomycin, penicillin and RPMI 1640 medium were purchased from WELGENE Inc. (Daegu, Republic of Korea). Antibodies against mouse TNF-α (cat# 109,828), mouse IL1b, mouse IL6 and mouse β-actin were purchased from BioLegend (San Diego, CA, USA).

### Preparation and characterization of glycol chitosan nanoparticles (CNPs)

Glycol chitosan nanoparticles (CNPs) were prepared by conjugation of 5β-cholanic acid to the primary amine groups of the glycol chitosan (GC). Briefly, 5β-cholanic acid (375 mg, 1 mmol), NHS (180 mg, 1.5 mmol), EDC (300 mg, 1.5 mmol) and GC (1.25 g, 2.5 μmol) were dissolved in distilled water/methanol mixture (1:1 v/v, 125 ml). The resulting solution was stirred for 12 h at 37 °C, followed by dialysis for 3 days in distilled water/methanol mixture (1:3 v/v) using a cellulose membrane (MWCO: 12,000–14,000). Then, the solution was further dialyzed for a day in distilled water and lyophilized to yield glycol chitosan-5β-cholanic acid conjugates. The near-infrared (NIRF) fluorescence dye, Cy5.5, was additionally introduced in CNPs. Briefly, Cy5.5-NHS (2 mg, 1.7 μmol) was dissolved in 1 ml of DMSO and added dropwise to the CNPs (220 mg) in DMSO solution. The reaction buffer was stirred for 48 h at 37 °C, dialyzed in the distilled water condition using a cellulose membrane (MWCO: 12,000–14,000) for a day, and lyophilized for 3 days to obtain Cy5.5-CNPs. Then, it was dispersed in distilled water or mouse serum, followed by analysis of size distribution using a Zetasizer Nano ZS (Malvern Instruments, Worcestershire, U.K.). The morphology of CNPs were observed by using transmission electron microscopy (TEM) (CM-200, Philips, CA, USA).

### Cellular uptake and cytotoxicity of CNPs

The cellular uptake of Cy5.5-CNPs was investigated in three types of normal cells, such as breast cancer cells (4T1), rat cardiomyocytes (H9C2), mouse fibroblasts (L929) and macrophages (Raw264.7). Each cell was incubated with Cy5.5-CNPs (100, 225 or 900 μg/ml) for 4 or 24 h. After treatment, cells were fixed with 4% paraformaldehyde for 15 min, and stained with 4′,6-diamidino-2-phenylindole (DAPI) for 10 min. The cellular uptake was observed using a Leica TCS SP8 confocal laser-scanning microscope (CLSM; Leica Microsystems GmbH; Wetzlar, Germany). Quantitative analyses of the fluorescence images were performed using ImageJ software (NIH, Bethesda, MD, USA). The cytotoxicity was assessed by the Cell Counting Kit-8 (CCK-8) assay. Briefly, 5 × 10^4^ 4T1, H9C2, L929 and Raw264.7 cells were seeded into 96-well cell culture plates. Then, each cell was treated with different concentration of Cy5.5-CNPs ranging from 0 to 900 μg/ml. After 24 h of incubation, the cells were further incubated with cell culture medium containing 10% of CCK-8 solution for 20 min. Finally, the cell viability was analyzed using a microplate reader (VERSAmaxTM; Molecular Devices Corp., USA) with a wavelength of 450 nm.

### Biodistribution of CNPs in healthy mice

The 5-week male BALB/c mice were purchased from NaraBio (Gyeonggi-do, Republic of Korea). Mice were bred under pathogen-free conditions in the Korea Institute of Science and Technology (KIST). All experiments with animals were performed in compliance with the relevant laws and institutional guidelines of Institutional Animal Care and Use Committee (IACUC; approved number of 2020–123) in Korea Institute of Science and Technology (KIST). First, pharmacokinetic (PK) profiles were assessed after intravenous injection with Cy5.5-CNPs (10, 22.5 or 90 mg/kg). After treatment, blood samples were collected from mice by cardiac puncture after deep anesthesia at pre-determined times and centrifuged at 2200 rpm to obtain plasma, followed by analysis of fluorescence intensity using an IVIS Lumina Series III system (PerkinElmer, Waltham, MA, USA). The area under the curves (AUC) and half-life (*t*_1/2_) were calculated using a WinNonlin software. The pharmacodynamic (PD) profiles were also assessed in mice by ex vivo fluorescence imaging of major organ (liver, lung, spleen, kidney, brain and heart) after 6 or 72 h of injection with Cy5.5-CNPs (10, 22.5 or 90 mg/kg). The fluorescence intensities in the major organs were quantified using a Living Image software (PerkinElmer, Waltham, MA, USA).

### Toxicity study of repeated and high dose of CNPs treatment in healthy mice

The toxicity of repeated and high dose of CNPs treatment was assessed by histological and hematological analyses. Briefly, Cy5.5-CNPs (10, 22.5 or 90 mg/kg) were intravenously injected into BALB/c mice with single- or multi-dosage (three times). On day 7 after treatments, major organs (liver, lung, spleen, kidney, brain and heart) were collected from mice, and structural abnormalities in organ tissues were assessed by staining with H&E. In the case of hematological analyses, blood samples were collected from the mice on day 7 and centrifuged at 2200 rpm to obtain plasma. The following factors in blood samples were measured; alanine aminotransferase (ALT), blood urea nitrogen (BUN), alkaline phosphatase (ALP), aspartate Aminotransferase (AST), creatine kinase (CK) and troponin I. The cardiotoxicity by Cy5.5-CNPs was further analyzed after multiple-dosage. The heart tissues were collected from mice after treatment with 10, 22.5 or 90 mg/kg of Cy5.5-CNPs three times. The accumulation of Cy5.5-CNPs in heart tissues was observed using a Leica TCS SP8 confocal laser-scanning microscope. Collagen fiber in heart tissues were stained with Masson's trichrome. Briefly, heart tissues were incubated in Bouin's fixative for 30 min at 56 °C, and the nuclei were co-stained with Weigert's iron hematoxylin. Then, cytoplasm was stained with Biebrich scarlet-acid fuchsin, and then differentiated in phosphomolybdic–phosphotungstic acid. The collagen matrix in heart tissues was stained with aniline blue solution. The collagen in heart tissues were quantitatively analyzed using an Image Pro software, and collagen contents were presented in proportion to the total area of heart tissues.

### Statistics

The statistical significance between two groups was analyzed using Student’s t-test. One-way analysis of variance (ANOVA) was performed for comparisons of more than two groups, and multiple comparisons were analyzed using the Tukey–Kramer post hoc test. Survival data was plotted as Kaplan–Meier curves and analyzed using the log-rank test. The statistical significance was indicated with asterisks (*p < 0.05, **p < 0.01, ***p < 0.001) in the figures.

## Supplementary Information

**Additional file 1: Figure S1.** Synthetic route to prepare the glycol chitosan and 5β-cholanic acid conjugates. **Figure S2.** Detail information of average size of different concentrations of CNPs in the mouse serum. **Figure S3.** Detail information of average size of different concentrations of CNPs in the mouse serum (n=5). **Figure S4.** Flow cytometric results showing H9C2 cells stained with Annexin V/PI after treatment with CNPs for 24 h. **Figure S5.** Fluorescence image of major organs from mice treated with 90 mg/kg of CNPs for 7 days. Fluorescence intensities were normalized with the results of Figure 3B and 3C. **Figure S6.** Excretion profile of Cy5.5-CNPs after 90 mg/kg treatment. The urines were collected from the mice at the indicated time points, followed by analysis of Cy5.5 fluorescence intensity using HPLC. **Figure S7.** Detail information of hematological parameters on day 7 after single- or multi-dose of 10, 22.5 or 90 mg/kg CNPs. **Figure S8.** Detail information of complete cell count results on day 7 after single- or multi-dose of 10, 22.5 or 90 mg/kg CNPs (n=5). **Figure S9.** Uncropped images of western blot results in Figure 5E.

## Data Availability

All relevant data are available with the article and its supplementary information files, or available the corresponding authors upon reasonable requests.
