# Supplementary material for: In vivo toxicity evaluation of tumor targeted glycol chitosan nanoparticles in healthy mice: repeated high-dose of glycol chitosan nanoparticles potentially induce cardiotoxicity
Source: J Nanobiotechnology. 2023 Mar 9;21:82. doi: 10.1186/s12951-023-01824-3 (PMC9999623; doi:10.1186/s12951-023-01824-3)
Supplement: Supplementary file 1 — Additional file 1: Figure S1. Synthetic route to prepare the glycol chitosan and 5β-cholanic acid conjugates. Figure S2. Detail information of average size of different concentrations of CNPs in the mouse serum. Figure S3. Detail information of average size of different concentrations of CNPs in the mouse serum (n=5). Figure S4. Flow cytometric results showing H9C2 cells stained with Annexin V/PI after treatment with CNPs for 24 h. Figure S5. Fluorescence image of major organs from mice treated with 90 mg/kg of CNPs for 7 days. Fluorescence intensities were normalized with the results of Figure 3B and 3C. Figure S6. Excretion profile of Cy5.5-CNPs after 90 mg/kg treatment. The urines were collected from the mice at the indicated time points, followed by analysis of Cy5.5 fluorescence intensity using HPLC. Figure S7. Detail information of hematological parameters on day 7 after single- or multi-dose of 10, 22.5 or 90 mg/kg CNPs. Figure S8. Detail information of complete cell count results on day 7 after single- or multi-dose of 10, 22.5 or 90 mg/kg CNPs (n=5). Figure S9. Uncropped images of western blot results in Figure 5E. [file 12951_2023_1824_MOESM1_ESM.docx]

**Additional file for**

*In vivo* toxicity evaluation of glycol chitosan nanoparticles in healthy mice: Repeated high-dose of glycol chitosan nanoparticles potentially induce cardiotoxicity

Hyeyoun Chang^1,#^, Ji Young Yhee^1,#^, Sangmin Jeon^1^, Man Kyu Shim^1^, Hong Yeol Yoon^1^, Sangmin Lee^2,*^ and Kwangmeyung Kim^1,3,*^

^1^ Medicinal Materials Research Center, Biomedical Research Division, Korea Institute of Science and Technology, Seoul, 02792, Republic of Korea.

^2^Department of Pharmacy, College of Pharmacy, Kyung Hee University, Seoul 02447, Republic of Korea.

^3^College of Pharmacy, Graduate School of Pharmaceutical Sciences, Ewha Womans University, Seoul 03760, Republic of Korea.

^#^These authors contributed equally to this work.

^*^Correspondence and requests for materials should be addressed to **K. Kim** (kimkm@ewha.ac.kr) and **S. Lee** (leesm@khu.ac.kr).

**
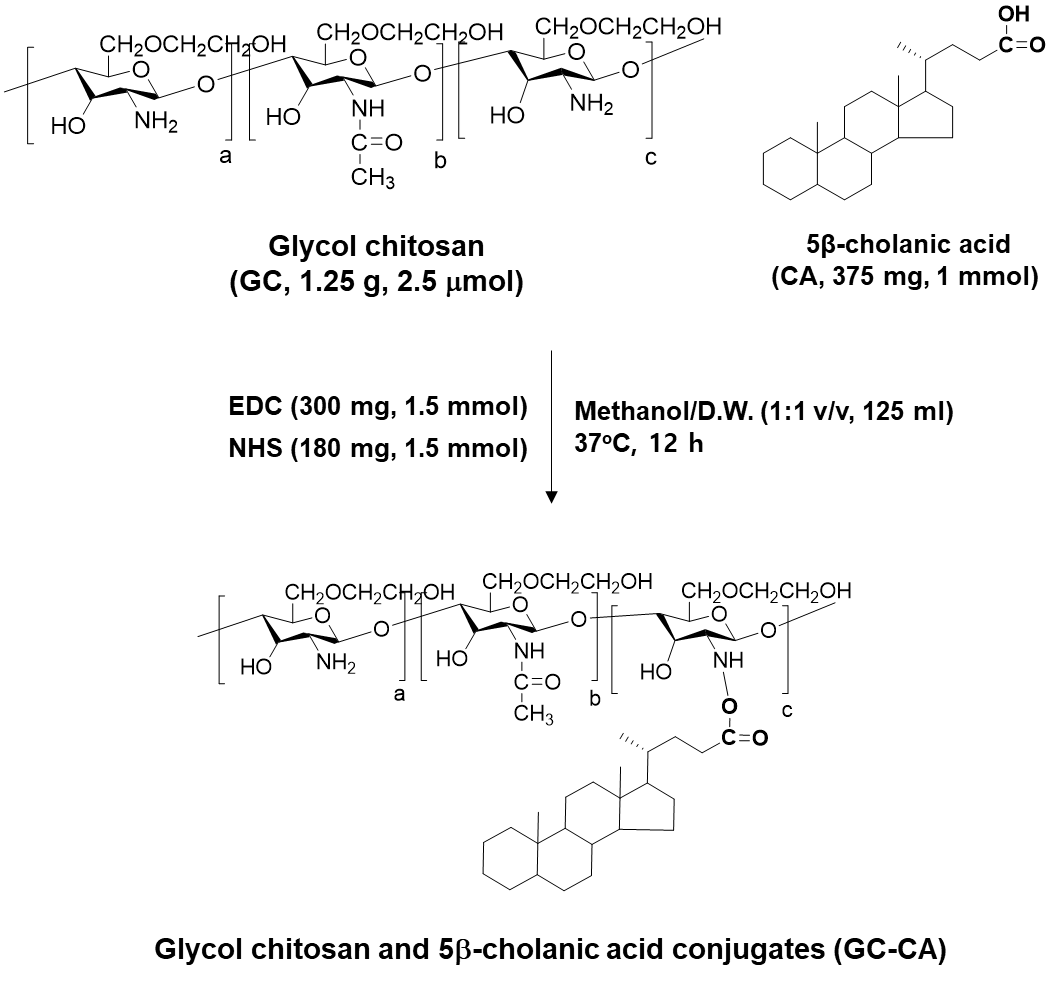
**

**Figure S1.** Synthetic route to prepare the glycol chitosan and 5β-cholanic acid conjugates.

**
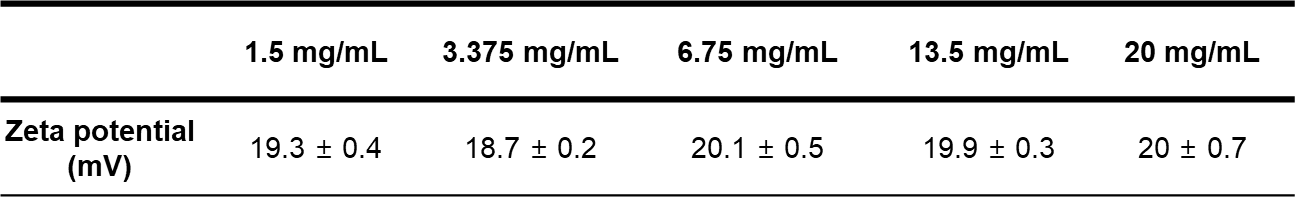
**

**Figure S2.** Zeta potential (mV) of CNPs in the clinically relevant highly concentrated aqueous condition (1.5, 3.375, 6.75, 13.5 or 20 mg/1 ml).


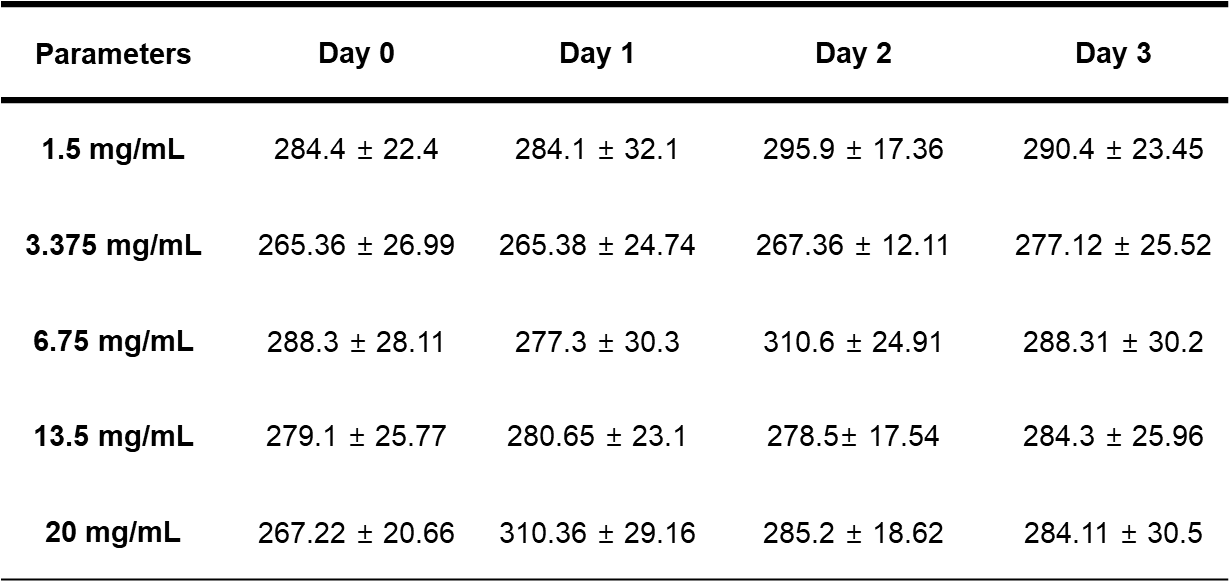


**Figure S3.** Detail information of average size of different concentrations of CNPs in the mouse serum (n=5).


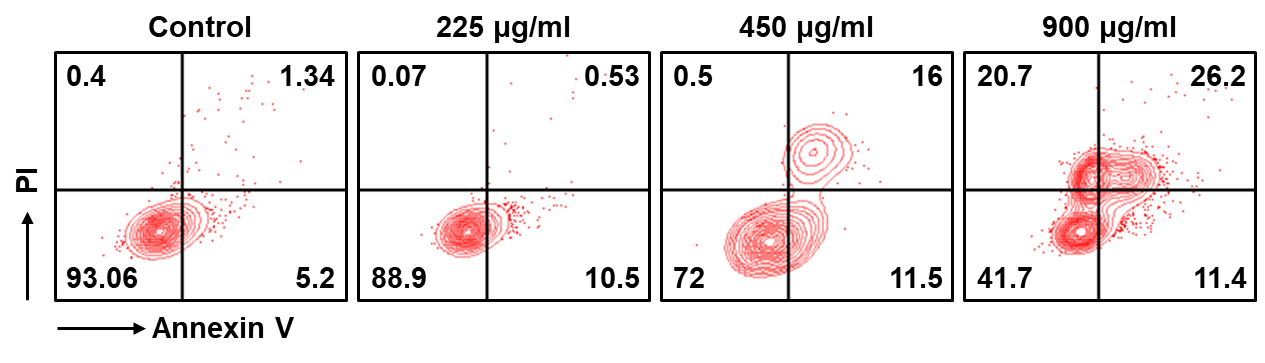


**Figure S4.** Flow cytometric results showing H9C2 cells stained with Annexin V/PI after treatment with CNPs for 24 h.


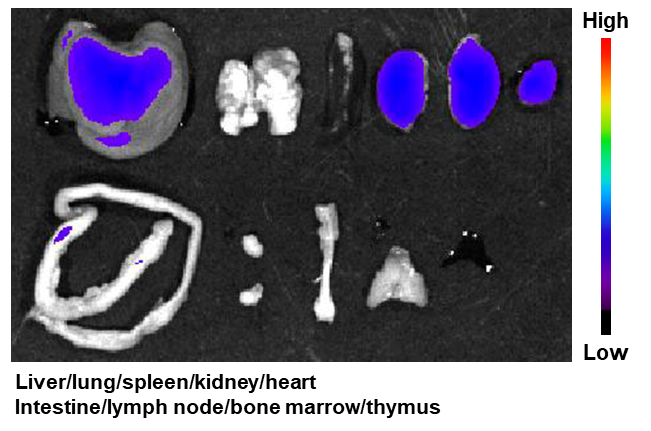


**Figure S5.** Fluorescence image of major organs from mice treated with 90 mg/kg of CNPs for 7 days. Fluorescence intensities were normalized with the results of Figure 3B and 3C.

**Figure S6. Excretion profile of Cy5.5-CNPs after 90 mg/kg treatment.** The urines were collected from the mice at the indicated time points, followed by analysis of Cy5.5 fluorescence intensity using HPLC.

**
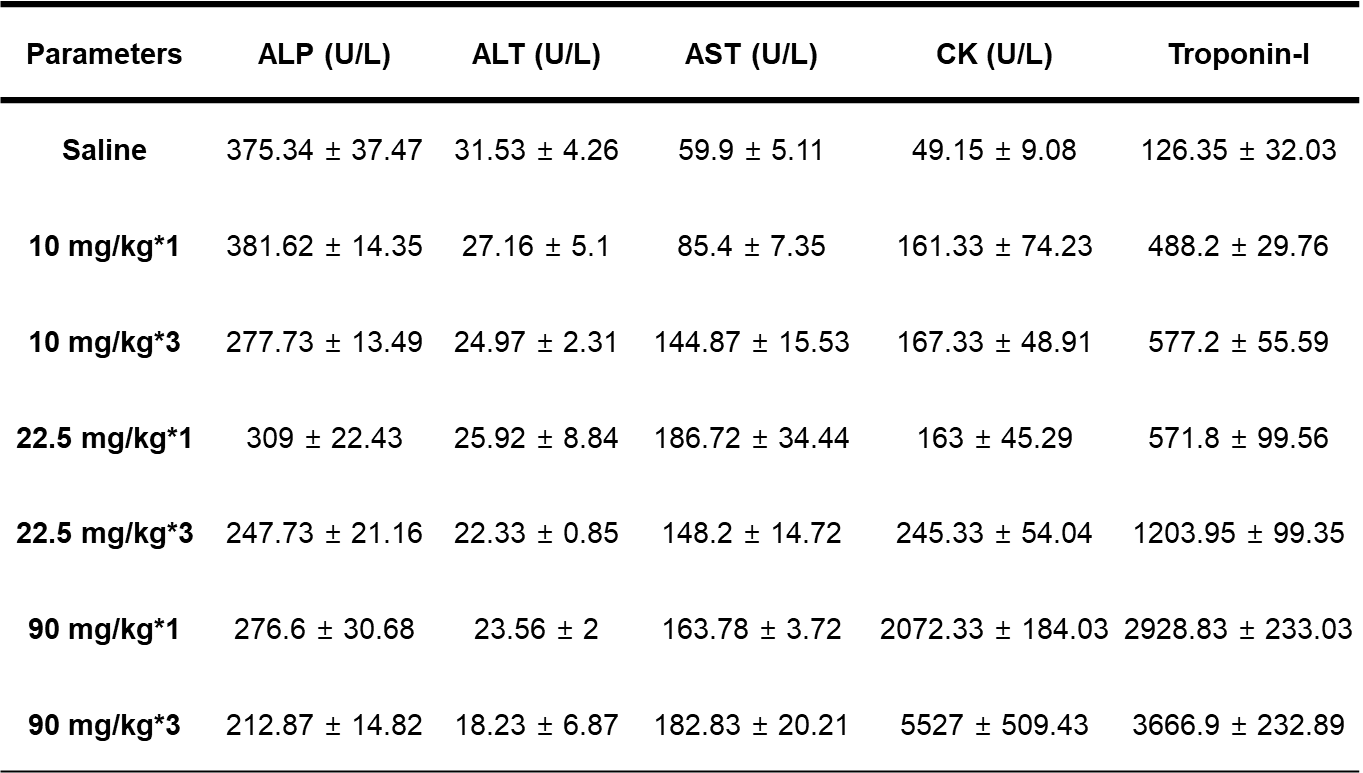
**

**Figure S7.** Detail information of hematological parameters on day 7 after single- or multi-dose of 10, 22.5 or 90 mg/kg CNPs (n=5).

**
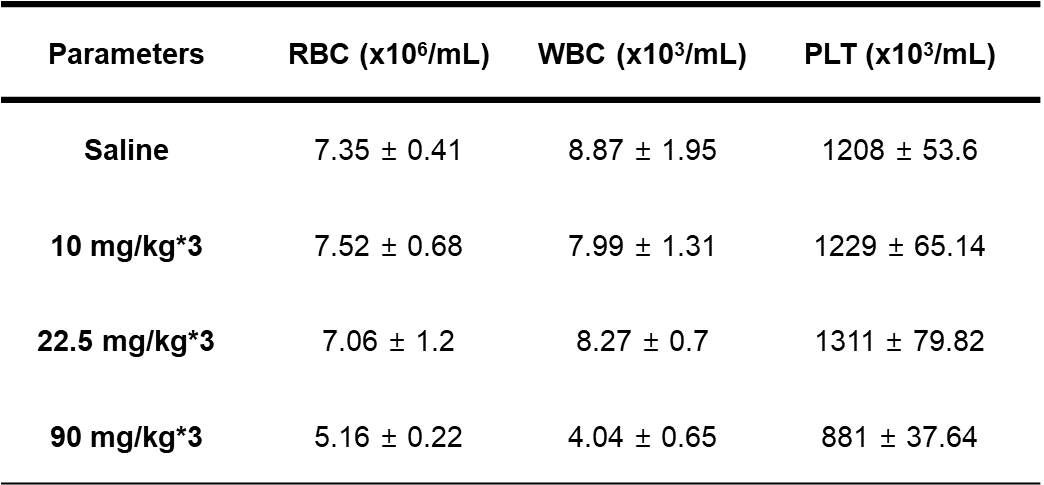
**

**Figure S8.** Detail information of complete cell count results on day 7 after single- or multi-dose of 10, 22.5 or 90 mg/kg CNPs (n=5).


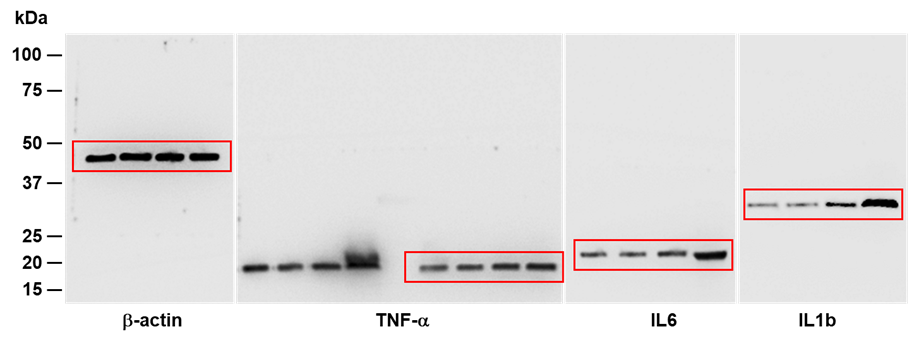


**Figure S9.** Uncropped images of western blot results in Figure 5E.
